# Antifungal Activity of Decyl Gallate against Several Species of Pathogenic Fungi

**DOI:** 10.1155/2014/506273

**Published:** 2014-11-20

**Authors:** Ana Carolina Alves de Paula e Silva, Caroline Barcelos Costa-Orlandi, Fernanda Patrícia Gullo, Fernanda Sangalli-Leite, Haroldo Cesar de Oliveira, Julhiany de Fátima da Silva, Liliana Scorzoni, Nayla de Souza Pitangui, Suélen Andrea Rossi, Tatiane Benaducci, Vanessa Gonçalves Wolf, Luis Octávio Regasini, Maicon Segalla Petrônio, Dulce Helena Siqueira Silva, Vanderlan S. Bolzani, Ana Marisa Fusco-Almeida, Maria José Soares Mendes-Giannini

**Affiliations:** ^1^Departamento de Análises Clínicas, Faculdade de Ciências Farmacêuticas, Universidade Estadual Paulista Júlio Mesquita Filho (UNESP), Rodovia Araraquara-Jaú, km 1, 14 800 901 Araraquara, SP, Brazil; ^2^Departamento de Química Orgânica, Instituto de Química, Universidade Estadual Paulista Júlio Mesquita Filho (UNESP), Rua Professor Francisco Degni, 55, Bairro Quitandinha, 14800-060 Araraquara, SP, Brazil

## Abstract

This work aims to demonstrate that the gallic acid structure modification to the decyl gallate (G14) compound contributed to increase the antifungal activity against several species of pathogenic fungi, mainly, *Candida* spp., *Cryptococcus* spp., *Paracoccidioides* spp., and *Histoplasma capsulatum*, according to standardized microdilution method described by Clinical Laboratory Standard Institute (CLSI) documents. Moreover this compound has a particularly good selectivity index value, which makes it an excellent candidate for broad-spectrum antifungal prototype and encourages the continuation of subsequent studies for the discovery of its mechanism of action.

## 1. Introduction

In the last two decades, there has been a rapid increase in the incidence of invasive fungal infections (IFIs) caused by fungal pathogens with diminished susceptibility or resistance to many standard antifungal agents. The early treatment of IFIs is essential for optimal clinical outcomes. The effectiveness of standard antifungal drugs (polyenes, azoles, and echinocandins) is not predictable against some emerging fungi and may cause undesirable side effects. Furthermore, the use of antifungals is often inappropriate, exposing patients to adverse effects, drug interactions, and the development of resistance to and super infections by other fungi, reducing their effectiveness and resulting in significant health expenditures. All of these factors are particularly problematic for immunocompromised (IC) or hospitalized patients with serious underlying diseases [1–5]. Considering that the diagnosis of these diseases remains challenging and that treatment is suboptimal, it is difficult to identify and implement the correct therapies.

The use of modified substances from natural compounds as prototypes of molecules for the treatment of diseases has increased abundantly in recent years. However, many of these products have no proven efficacy and safety. The aim of this study was adding new results about the antifungal activity* in vitro* of 14 alkyl gallates against important pathogenic fungi, mainly,* Candida parapsilosis*,* C. krusei*,* Cryptococcus gattii*,* Histoplasma capsulatum*, and* Paracoccidioides* spp. The literature presents several data about a wide range of biological activities, and its chemical structures have correlation with the antifungal activity and the cytotoxicity [6, 7]. Kubo et al. [8] made an important observation about the length and hydrophobicity of alkyl groups; to a large extent, these factors are associated with their antifungal activity. Because of the wide range of properties and commercial applications, alkyl gallates are compounds of great interest to both pharmaceutical and chemical industries [9]. Besides, we highlighted the decyl gallate role as a promising broad-spectrum antifungal.

## 2. Materials and Methods

### 2.1. Microorganisms

*C. albicans* ATCC 90028 (Ca),* C. krusei* ATCC 6258 (Ck),* C. parapsilosis* ATCC 22019 (Cp),* C. neoformans* ATCC 90012 (Cn), and* C. gattii* ATCC 56990 (Cg) were selected for the study. Four filamentous species were also used, including* T. mentagrophytes* ATCC 11481 (Tm),* T. rubrum* ATCC 28189 (Tr),* A. fumigatus* ATCC 7100 (Af), and* A. niger* ATCC 16404 (An). All species were obtained from the collection of Clinical Mycology Laboratory, School of Pharmaceutical Sciences, UNESP, Araraquara, São Paulo, Brazil. This study also included the dimorphic fungi* H. capsulatum* var.* capsulatum* EH-315 strain (Hc);* P. brasiliensis* isolates 18, D03, and 339 (belonging to the S1 phylogenetic species), isolate 02 (PS2 phylogenetic species), and isolate Epm83 (PS3 phylogenetic species); and* P. lutzii* strain 01 (ATCC MYA-826) and two isolates, EE and 8334MMT (origin is described in Acknowledgments).

### 2.2. Susceptibility Tests

The minimum inhibitory concentration (MIC; mg L^−1^) was determined by the antifungal susceptibility test for all species, following the reference broth microdilution method, as outlined in the CLSI. The M27-A3 document [10] was used for yeast and dimorphic fungi species. For dimorphic fungi microdilution test was performed according to de Paula e Silva et al. [11]. The M38-A2 document [12] was used for filamentous species. The determination of the minimum fungicidal concentration (MFC; mg L^−1^), which is the lowest concentration that did not allow the growth of any fungal colony on the solid medium after the incubation period, was performed as was done by Regasini et al. [13] and Gullo et al. [14]. For this an aliquot from the wells was transferred to a plate with Sabouraud medium (Sigma-Aldrich, St. Louis, MO, USA) and incubated at 37°C for the time determined for each species. All the tests were performed in triplicate and in three independents assays. The strain* C. krusei* ATCC 6258 was used also as a quality control for both tests.

The following antifungal drugs were used as controls: amphotericin B (AMB), itraconazole (ITZ), fluconazole (FLZ), terbinafine (TERB), and griseofulvin (GRIS; Sigma-Aldrich, St. Louis, MO, USA). All antifungal drugs were diluted according to the instructions of each CLSI document. The final dilutions and inoculum were in RPMI 1640 medium with L-glutamine without bicarbonate (Gibco; Grand Island, NT, USA) buffered to pH 7.0 with 0.165 M 3-*N*-morpholinopropanesulfonic acid (Sigma-Aldrich, St. Louis, MO, USA) with 2% glucose.

### 2.3. Preparation of Alkyl Gallates

Alkyl gallates (Table 1) were synthesized as previously described by Morais et al. [15]. Five milligrams of each dried substance was diluted and solubilized aseptically in appropriate quantities of dimethylsulfoxide (DMSO; Sigma-Aldrich, St. Louis, MO, USA). The amount of DMSO used was previously tested and did not affect the fungal viability (data not shown). For the experiments, the concentration of each substance was calculated for a range of concentrations from 62.5 to 0.002 mg L^−1^ by dilutions in RPMI 1640 medium in a microdilution plate; then the test was performed in accordance with the M27-A3 and M38-A2 documents.

### 2.4. Cytotoxicity Tests

The cellular cytotoxicity of the alkyl gallates was evaluated against lung tumor cells (A549) and normal fibroblast pulmonary cells (MRC-5), which were obtained from the American Type Culture Collection (Manassas, VA, USA). The cytotoxicity test was performed by MTT [16] using 3-(4,5-dimethyl-2-thiazolyl)-2,5-diphenyl-2*H*-tetrazolium bromide (Sigma-Aldrich, St. Louis, MO, USA) at 5 mg mL^−1^. Spectrophotometric readings were taken from microplates in an ELISA reader (Bio-Rad model 3550) at a wavelength of 540 nm. Untreated cells constituted the positive control (viable cells), and cells treated with hydrogen peroxide (Sigma-Aldrich, St. Louis, MO, USA) constituted the negative control (death cells). All the tests were performed in triplicate in three independents assays. A test was performed on plates without cells to verify that the reaction cannot occur between alkyl gallates and the reagent to avoid false-positive results (data not shown). Statistical analysis was performed using 2-way ANOVA with Bonferroni post-test using GraphPAd Prism 5 software. *P* values > 0.05 were considered statistically not significant in relation to the death control.

The IC_50_ values of both cell lines were calculated in relation to G1 molecule and the best antifungal activity of alkyl gallate is the G14. This value represents the concentration required for 50% cell death (i.e., the concentration of each alkyl gallate that results in 50% absorbance reduction compared with untreated cells, termed the IC_50_) [17]. The selective index (SI) was calculated to both cells lines, which is defined as the ratio of the measured IC_50_ in the two cell lines to the MIC of the tested alkyl gallate (i.e., SI = IC_50_/MIC). The SI was considered significant when >10 [18, 19].

## 3. Results

Table 2 shows the MICs of gallic acid (G1) and the alkyl gallates. These molecules correspond to esters with a different number of carbon chains (G2 to G17, as shown in Table 1). Depending on the fungi, the G1 MIC value varied from 4 to 62.5 mg L^−1^ and the same results were observed for G2 to G11. In general, from the alkyl gallates G12 to G15, there was a decrease in the MIC, ranging from 2 to 16 mg L^−1^, to the majority of fungal species, except to* Aspergillus sp.* and* C. krusei,* which presented value of 31 and >62.5 mg L^−1^. There was an increase in the MIC value to the alkyl gallates G16 and G17. For the genus* Paracoccidioides*, G12 to G15 presented the lowest MICs for the majority of isolates (0.004 to 0.5 mg L^−1^), and the G16 showed low MIC values (0.015 to 0.125 mg L^−1^). The MIC value to* H. capsulatum* was 2 mg L^−1^ for the G12 to G15 alkyl gallates.

Some of the best MICs of the alkyl gallates were similar to or lower than the MICs found for current therapeutic antifungal agents.* C. krusei* and* T. rubrum* are FLZ resistant strains with MIC of 64 mg L^−1^ to these agents, and the G12 and G14 presented MICs of 4–8 mg L^−1^. Most of the alkyl gallates showed MFC values similar to the MIC values. Nevertheless, G14 had the best MIC when it was evaluated against most fungal species.

**Figure 1:**
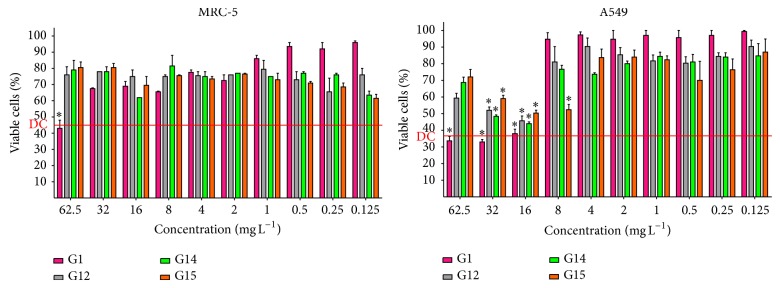
Cell viability tested in MRC-5 and A549 after treatment with different concentrations of gallic acid (G1), octyl gallate (G12), decyl gallate (G14), and undecyl gallate (G15). ∗ Indicates that there was no difference statistic (*P* > 0.05) in relation death control (red line represents the mean percentage of viable cells in the death control (DC)).

*The cytotoxicity of gallic acid and the alkyl gallates *were* evaluated in respiratory* epithelial cells showing high cell viability to MRC5 and A549 cells line, revealing the low cytotoxicity of these compounds to both cell lines.  Figure 1 shows the results of the cytotoxicity test when the cell lines were treated with G1 and the alkyl gallates G12, G14, and G15, that is, those that had the best antifungal activity against most fungi species tested in this study, and we could observe that these selected alkyl gallates showed low cytotoxicity to both cell lines. In this test, the viability percentage is considered satisfactory when it is above the mean percentage of death control. In Supplementary Material we present the results of the cytotoxicity test to all alkyl gallates used in this study (see Supplementary Material available online at http://dx.doi.org/10.1155/2014/506273).

Table 3 presents the IC_50_ values of A549 and MRC-5 cell lines for G1 and G14. These alkyl gallates showed IC_50_ values above the MICs and MFCs determined for the different fungi. The SI was also demonstrated in Table 3, and we could observe that G14 showed the greatest antifungal activity against the majority of fungal species and had higher SI values for both the MRC-5 and A549 cell lines.

## 4. Discussion

Gallic acid (GA) or 3,4,5-trihydroxybenzoic acid is a natural plant triphenol and it can be produced by acid hydrolysis of tannic acid. The substitution of the GA acid portion allows the obtainment of analogues esters with distinct physicochemical characteristics, especially lipophilicity that is evaluated by the partition coefficient, called alkyl gallates, according to the atom carbon number in the side chain [6, 7]. GA acts as an antiapoptotic agent and protects human cells against oxidative damage, as it has the ability to scavenge and reduce reactive oxygen species (ROS) formation [20]. The alkyl gallates, like methyl, propyl, octyl, and dodecyl gallates, have a wide range of biological activities, used in food manufacturing as antioxidants, as well as in the pharmaceutical and cosmetic industries, as well as antifungal [6, 21], antibacterial [22], antiviral [23], antitumoral [7], and antihemolytic activities [24]. Because of these several interesting properties and commercial applications, alkyl gallates are compounds of great interest to both pharmaceutical and chemical industries.

Our group showed MIC values equivalent to the values found by Leal et al. [6], which confirm the reproducibility of antifungal activity of the alkyl gallates, and brings new results with other important pathogenic fungi, as* C. parapsilosis*,* C. krusei*,* C. gattii*,* H. capsulatum*, and* Paracoccidioides* spp. Current increases of antifungal drug resistance in* Candida* spp. and clinical treatment failures are of concern, as invasive candidiasis is a significant cause of mortality in intensive care units [25]. Cryptococcosis is an important globally infectious disease. The majority of illness is among patients with defective cell-mediated immunity. The most common clinical presentation is* Cryptococci* meningitis, with over 1 million cases and 600,000 deaths per year [26]. Between the most commonly endemic mycoses described are paracoccidioidomycosis and histoplasmosis; both are difficult to diagnose, because of limiting factors as isolation conditions and sensitivity and specificity of microscopic examination of fluids and tissues which does not lead to immediate diagnosis, hindering successful subsequent treatments [27]. These difficulties constitute serious problems and underscore the need for a better understanding of the pathophysiology of different fungal infections, which could help to identify new targets of drug therapy and lead to the development of new antifungal agents [2, 3].

Then, to evaluate the susceptibility to gallic acid and alkyl gallates, 18 fungi species were assayed. G1 showed high MIC value for most isolates and the same results were observed for G2 to G11, suggesting that the esterification process in the original G1 molecule did not improve the antifungal activity. However, the esterification process for the formation of G12 to G15 showed MICs values significantly different; in other words, chains with eight to eleven carbons enhanced the antifungal activity. These findings indicate that this modification contributed positively to improvement of the antifungal activity of the compound. However, this pattern was not observed in G16 and G17 against most isolates. Instead, higher MICs values were recorded, suggesting a cutoff limit for the chain size.

There was not a change in the susceptibility among the alkyl gallates that presented antifungal activity and the species of same genus, but the MIC value found was considered low. This pattern was observed for* Candida* and* Cryptococcus* species, important yeasts more frequently in the last years in critically ill patients [25, 26]. These results confirm the hypothesis that these compounds can be better studied to find the unique compound against the majority of clinical importance fungi.

*Histoplasma capsulatum* showed constant MIC values to G12 to G15, which indicate that the chains with eight to eleven carbons have the same effect on this fungus, and the* Paracoccidioides* genus is more susceptible to alkyl gallates than the other fungal species tested. These results show that these compounds can be effective in cases of therapies with difficult diagnosis of specific species or genus. High susceptibility between the species* P. lutzii* and* P. brasiliensis* was observed suggesting the necessity of more studies about the mechanism action involved.

We agree with the conclusion presented by Leal et al. [6] that the activity can vary with the fungi tested, mainly between groups of fungi, and that the antifungal activity of alkyl gallates appears to be dependent on the presence of a catechol moiety along with a hydrophobic alkyl chain, similar to the activity of alkanols described by Kubo et al. [28].

Several studies suggest the mechanism of action involves these compounds. Fujita and Kubo [29] suggested by glucose-induced medium acidification method [30] that an alkyl gallate with nine carbons in the chain side causes damage in the cellular membrane including the plasma membrane, because this compound has three hydrophilic hydroxyl groups in the head of gallate acids and the hydrophobic alkyl chain in the tail, disturbing the stable structure of lipid membrane bilayers. The probable explanation is that hydroxyl groups would interact with hydrophilic groups thrusting on the membrane to form hydrogen binds. The nonpolar carbon chains are folded into the membrane bilayers, resulting in a change of the membrane fluidity. This injury probably resulted in the leakage of potassium ions from fungi cells. This study also suggested that this alkyl gallate might achieve inner membrane of mitochondria, influencing mitochondrial functions related to ROS generation.

The development of an antifungal agent is considered challenging because potential targets can be shared by yeast and mammalian cells; both are eukaryotes and have some homologous metabolic pathways. The optimal antifungal agent should have a wide activity spectrum, have a fungicidal action rather than fungistatic action, be available for oral and parenteral use, be safe in the efficacious dose, have a great cost-effectiveness, and be stable to microbial resistance. Considering all these points, novel and selective molecular targets for the development of new antifungal agents with the goal of minimizing toxicity are of great importance.

In this sense,* in vitro* cytotoxicity assays are the first step to assess whether a compound with potential antifungal activity is promising to become a future antifungal agent. In this study, two lung cell lines were used for the cytotoxicity by MTT assay, since the majority of fungi tested are bound to the respiratory tract. Then, the cell viability for both lines remained greater than 50% at most of concentrations of G14 and the other two alkyl gallates, G12 and G15, considered with good antifungal activity, by the MTT assay.

MTT assay detects the decrease of viable cells number by the reduction of tetrazolium salt to formazan into living cells that occurs mainly through electron transfer at the mitochondrial level [16, 31]. There are in the literature several reports about the cytotoxicity of alkyl gallates. Locatelli et al. [7] described in a review that alkyl gallates are not cytotoxic against rat liver slices and/or nontumoral cell line (monkey kidney fibroblasts, VERO cells), normal mouse brain endothelial cells, human lymphocytes, or when administered in rats or mice.

Another factor to consider is the detachable selectivity index. The SI is an indication of the relative safety of a compound, where higher SI values reflect greater safety, once that this value is defined as the ratio of the IC_50_ for the MIC value. In other words, the necessary concentration of the alkyl gallate to kill 50% of the health mammalians cells is higher than the concentration considered fungicide. Then SI will be greater than 10 because the values are inversely related. The ratio between the safety and potency of a compound is a very important parameter to consider in the development of therapeutic products [17, 32, 33]. Accordingly, the alkyl gallate G14 had better MIC values and presented an SI above 10 against most fungi, revealing that this alkyl gallate is safe. These data suggest the importance of more studies about the difference of action mechanism between the fungal and mammalian cells, searching for the explanation of the fact that an alkyl gallate with a ten-carbon chain had better activity than the others, with one carbon to more or less.

A point that should be emphasized in favor of this compound is that most of MIC values were similar to or lower than the values of standard antifungal agents, indicating the importance of these substances as promising antifungal agents. One of the major obstacles to antifungal therapy is toxicity, the associated high cost, the development of intrinsic resistance, or a reduced susceptibility to available antifungal agents.

Amphotericin B is considered the “gold standard” to mainstay of antifungal therapy because of its broad-spectrum activity and few cases of mycological resistance. However, it is associated with frequent and potentially serious adverse effects. The serious adverse effects led to the development of AmB formulations, which presented lower rates of nephrotoxicity, but the cost of these agents is substantial, and access is limited in resource-limited settings [34, 35]. On the other hand, the recent increases in* Candida* spp. resistance to echinocandins and azoles have led to clinical failures. This is a matter of concern because of the limited number of drug classes targeting different fungal components and because the number of patients at risk receiving treatment is continually growing, thus further increasing antifungal drug pressure [25]. In this sense, besides of the possibility of a treatment isolate with this alkyl gallate, a combinatory therapy of the alkyl gallate with the available antifungals agents can be an option that can lead to reduce the dose or improving the action of both antifungal agents.

In conclusion, in this preliminary study, among a series of 14 alkyl gallates, our group selected the decyl gallate that was considered to have the best antifungal activity for a wide variety of pathogenic fungi with clinical importance. These suggest how important the structure activity relationship is to obtain the best antifungal performance. It is a promising compound for further studies once the cytotoxicity tests were compatible with those already described in the literature, and the fungicide action mechanism is not prejudice to mammals cells, showing a SI that guarantees the safety required in this initial step.

## Supplementary Material

The Supplementary Material show the cell viability of MRC-5 and A549 cell lines when in contact with ten different concentrations of the gallic acid and of the 14 alkyl gallates. ∗ Indicates that there was no difference statistic (p > 0.05) in relation death control (red line represents the mean percentage of viable cells in the death control (DC)).

## Figures and Tables

**Table 1:** Molecular structure of gallic acid (G1) and alkyl gallates (G2–G17).

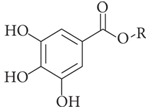			

R	G1	Gallic acid	H
	G2	Methyl gallate	CH_3_
	G3	Ethyl gallate	CH_2_CH_3_
	G4	Propyl gallate	(CH_2_)_2_CH_3_
	G5	Isopropyl gallate	CH(CH_3_)_2_
	G6	Butyl gallate	(CH_2_)_3_CH_3_
	G7	Pentyl gallate	(CH_2_)_4_CH_3_
	G9	Isobutyl gallate	CH_2_CH(CH_3_)_2_
	G10	Hexyl gallate	(CH_2_)_5_CH_3_
	G11	Heptyl gallate	(CH_2_)_6_CH_3_
	G12	Octyl gallate	(CH_2_)_7_CH_3_
	G14	Decyl gallate	(CH_2_)_9_CH_3_
	G15	Undecyl gallate	(CH_2_)_10_CH_3_
	G16	Dodecyl gallate	(CH_2_)_11_CH_3_
	G17	Tetradecyl gallate	(CH_2_)_13_CH_3_

**Table 2:** MICs/MFCs of gallic acid and alkyl gallates against different fungal species.

	Ca	Ck	Cp	Cn	Cg	Tm	Tr	Afu	Ani	Hc	EE	8334MMT	01	18	D03	339	02	Emp83
G1	62.5/#	31/#	31/#	31/>62.5	31/>62.5	31/#	16/#	>62.5/#	>62.5/#	1/#	8/#	>62.5/#	4/#	16/#	>62.5/#	31/#	4/#	4/#
G2	>62.5/#	>62.5/#	>62.5/#	>62.5/#	16/>62.5	62.5/#	62.5/#	>62.5/#	>62.5/#	31/#	4/#	16/#	2/#	16/#	16/#	16/#	16/#	1/#
G3	>62.5/#	>62.5/#	>62.5/#	>62.5/#	16/>62.5	62.5/#	62.5/#	>62.5/#	>62.5/#	62.5/#	8/#	16/#	1/#	8/#	4/#	8/#	16/#	1/#
G4	>62.5/#	>62.5/#	>62.5/#	>62.5/#	16/>62.5	62.5/#	31/#	>62.5/#	>62.5/#	31/#	2/#	8/#	1/#	8/#	2/#	4/#	4/#	0.25/#
G5	>62.5/#	>62.5/#	>62.5/#	62.5/>62.5	16/>62.5	62.5/#	31/#	>62.5/#	>62.5/#	16/#	2/#	4/#	2/#	16/#	2/#	4/#	4/#	0.015/#
G6	>62.5/#	>62.5/#	>62.5/#	16/>62.5	8/>62.5	31/#	16/#	>62.5/#	>62.5/#	16/#	2/#	8/#	2/#	2/#	2/#	4/#	4/#	0.015/#
G7	>62.5/#	>62.5/#	>62.5/#	8/>62.5	4/>62.5	16/#	16/#	>62.5/#	>62.5/#	4/#	0.5/#	2/#	0.25/#	0.5/#	0.25/#	1/#	1/#	0.015/#
G9	>62.5/#	>62.5/#	>62.5/#	16/>62.5	4/>62.5	31/#	31/#	∗	∗	8/#	4/#	8/#	1/#	2/#	1/#	4/#	2/#	0.25/#
G10	62.5/#	31/>62.5	31/#	8/31	2/31	16/#	8/#	>62.5/#	>62.5/#	4/#	0.5/#	2/#	0.25/#	0.25/#	0.25/#	0.5/#	2/#	1/#
G11	16/31	31/#	31/#	2/16	1/16	4/#	8/#	62.5/#	31/#	2/#	0.125/#	0.25/#	0.125/#	0.125/#	0.125/#	0.03/#	0.5/#	0.25/#
G12	8/#	8/#	8/#	2/8	1/8	8/#	8/#	31/#	31/#	2/#	0.125/#	0.25/#	0.125/#	0.015/#	0.125/#	0.015/#	0.25/#	0.015/#
G14	4/#	4/31	4/#	1/4	1/2	4/#	8/#	31/#	8/#	2/#	0.03/#	0.125/#	0.125/#	0.004/#	0.125/#	0.004/#	0.004/#	0.008/#
G15	2/#	4/>62.5	2/4	1/4	0.5/4	4/#	4/#	>62.5/#	>62.5/#	2/#	0.03/#	0.03/#	0.125/#	0.015/#	0.125/#	0.008/#	0.03/#	0.015/#
G16	2/62.5	4/>62.5	4/16	1/4	1/4	4/#	4/#	>62.5/#	>62.5/#	4/#	0.03/#	0.03/#	0.125/#	0.125/#	0.125/#	0.06/#	0.015/#	0.5/#
G17	4/>62.5	4/>62.5	31/#	1/16	0.5/4	4/#	>62.5/#	>62.5/#	>62.5/#	>62.5/#	0.125/#	0.25/#	0.125/#	0.25/#	0.125/#	0.125/#	0.03/#	0.06/#
**AMB**	**1**	**2**	**0.5**	**0.25**	**0.5**	∗	∗	**8**	**4**	**0.03**	**0.25**	**0.25**	**0.06**	**0.125**	**0.008**	**0.06**	**0.125**	**0.06**
**ITZ**	**0.5**	**0.5**	**0.25**	∗	∗	∗	∗	**>16**	**>16**	**0.03**	**0.008**	**0.03**	**0.008**	**0.015**	**0.015**	**0.015**	**0.008**	**0.015**
**FLZ**	**1**	**64**	**2**	**4**	**4**	**1**	**64**	**∗**	**∗**	**0.125**	**∗**	**∗**	**∗**	**∗**	**∗**	**∗**	**∗**	**∗**
**TERB**	∗	∗	∗	∗	∗	**0.008**	**0.03**	**∗**	**∗**	∗	**∗**	**∗**	**∗**	**∗**	**∗**	**∗**	**∗**	**∗**
**GRIS**	∗	∗	∗	∗	∗	**0.5**	**1**	**∗**	**∗**	∗	**∗**	**∗**	**∗**	**∗**	**∗**	**∗**	**∗**	**∗**

^#^MFC = MIC, ^*^compound not tested for this species.

**Table 3:** The IC_50_ and SI values for both cell lines and all fungi species against gallic acid (G1) and decyl gallate (G14).

	SI			
	G1		G14	
	MRC-5	A549	MRC-5	A549
Ca	0	0	>10	2
Ck	0	0	2	>10
Cp	0	0	>10	>10
Cn	0	0	>10	>10
Cg	0	0	>10	>10
Tm	0	0	>10	>10
Tr	0	0	6	9
Af	0	0	2	2
An	0	0	6	9
Hc	3	2	>10	>10
EE	0	0	>10	>10
8334MMT	0	0	>10	>10
1	1	1	>10	>10
18	0	0	>10	>10
D03	0	0	>10	>10
339	0	0	>10	>10
2	1	1	>10	>10
Epm83	1	1	>10	>10

IC_50_ (mg L^−1^)	73	93	50	71
