# Supplementary material for: Antifungal Activity of Decyl Gallate against Several Species of Pathogenic Fungi
Source: Evid Based Complement Alternat Med. 2014 Nov 20;2014:506273. doi: 10.1155/2014/506273 (PMC4258339; doi:10.1155/2014/506273)

Supplementary Material

Cell viability tested in MRC-5 and A549 after treatment with different concentrations of alkyl gallates. The red line represents the mean percentage of viable cell in the death control (DC). *p > 0.05 not difference statistic in relation death control.

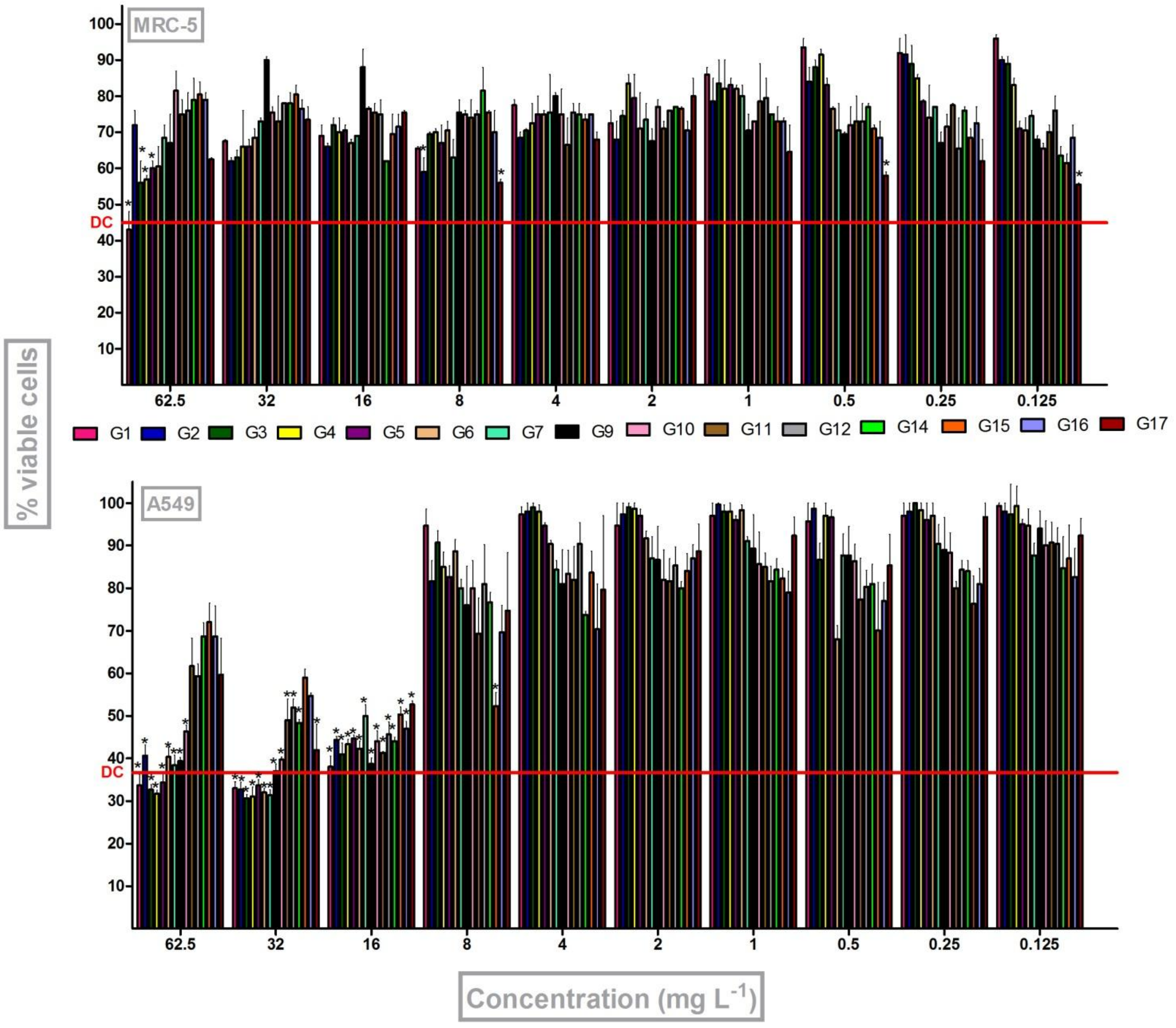

Supplement: Supplementary file 1 — The Supplementary Material show the cell viability of MRC-5 and A549 cell lines when in contact with ten different concentrations of the gallic acid and of the 14 alkyl gallates. ∗ Indicates that there was no difference statistic (p > 0.05) in relation death control (red line represents the mean percentage of viable cells in the death control (DC)). [file 506273.f1.pdf]
